# Neck circumference is associated with adipose tissue content in thigh skeletal muscle in overweight and obese premenopausal women

**DOI:** 10.1038/s41598-020-65204-9

**Published:** 2020-05-20

**Authors:** Maria Jose Arias Tellez, Analiza M. Silva, Jonatan R. Ruiz, Sandra S. Martins, António L. Palmeira, Teresa L. Branco, Claudia S. Minderico, Paulo M. Rocha, José Themudo-Barata, Pedro J. Teixeira, Luís B. Sardinha

**Affiliations:** 1 Department of Nutrition, Faculty of Medicine, University of Chile, Independence, 1027 Santiago, Chile; 2 PROFITH “PROmoting FITness and Health through physical activity” research group. Department of Physical and Sports Education, Sport and Health University Research Institute (iMUDS), Faculty of Sports Science, University of Granada, Ctra de Alfacar s/n C.P., 18071 Granada, Spain; 3 Exercise and Health Laboratory, CIPER, Faculdade Motricidade Humana, Universidade de Lisboa, Estrada da Costa, 1495-688 Cruz Quebrada, Portugal; 4 Universidade Europeia, Lisbon, Portugal; 5 Instituto de Saúde Ambiental (ISAMB), Faculdade de Medicina, Universidade de Lisboa, Lisbon, Portugal

**Keywords:** Obesity, Risk factors

## Abstract

Neck circumference (NC) has been proposed as a simple and practical tool, independently associated with cardiometabolic risk factors. However, the association of NC with inter-muscular adipose tissue (IMAT) is still to be determined. We aimed to examine the association of NC with thigh IMAT, and visceral adipose tissue (VAT) measured with computed tomography (CT) in overweight/obese women. 142 premenopausal overweight and obese Caucasian women participated in this cross-sectional study. NC was measured with an inextensible metallic tape above the thyroid cartilage according to International Society for Advancement of Kinanthropometry protocol. Thigh IMAT and VAT volumes were measured with a single cross-sectional CT. Regarding the covariates, fat mass (FM) was assessed with dual-energy x-ray absorptiometry and physical activity was objectively measured with accelerometry. NC was positively associated with thigh IMAT and VAT volumes (standardized β coefficient: β = 0.45, P-value = ≤0.001, β = 0.60, P = ≤ 0.001; respectively), which persisted after adjusting for age, height, overall FM or moderate-to-vigorous physical activity. Our findings show that NC is associated with thigh IMAT volume in overweight and obese premenopausal Caucasian women, regardless of the amount of lower-body fatness. These results suggest underscoring the relevance of NC as a marker of adipose tissue content in thigh skeletal muscle.

## Introduction

Obesity is associated with significantly higher all-cause mortality in almost two thirds of the adult population in developed countries^1^. Although the deleterious effects of greater adiposity are well documented, obesity-related metabolic consequences are more associated with regional body fat distribution, ectopic fat deposition, and cellular infiltration, rather than absolute quantity^2,3^. An increased upper-body fat has been associated with adverse metabolic complications, in particular the visceral adipose tissue (VAT) located in the thoracic, abdominal and pelvic cavities^4,5^. In addition, a higher intermuscular adipose tissue (IMAT) located between muscle groups and beneath the fascia^6^ is related to greater risk of all-cause mortality. Each one-standard deviation increase in IMAT (~6.8% greater IMAT) is associated with a 40% greater mortality risk over a 10-year period^7^. IMAT volume is higher in individuals with obesity and type 2 diabetes^8,9^ and is associated with high levels of inflammation, insulin resistance, obesity, sarcopenia, and lower physical performance in adults and the elderly population^10,11^. Recently, Bergia *et al*. showed that greater IMAT in the thigh is a better predictor of cardiometabolic risk than greater IMAT in the calf in adults who are overweight and obese^11^. Interestingly, IMAT is a unique ectopic adipose depot involved in a range of adverse health outcomes similar to VAT^12^.

In the body, adipose tissue two compartments are clearly distinguished: Subcutaneous adipose tissue (SAT) (80% of all body), and VAT (intra-abdominal fat)^13^. IMAT have been definite as part of SAT depot located between muscle groups and beneath the muscle fascia^14^. Advances in imaging technology have enabled the identification of IMAT and VAT thought the use of highly sensitive imaging techniques as computed axial tomography (CT) scan and magnetic resonance imaging (MRI)^15^.

Recently, emerged evidence reveals that volume of neck adipose tissue is independently associated with cardiovascular risk factors, metabolic syndrome prevalence and long-term mortality^16–20^. Consequently, neck circumference (NC) has been proposed as a novel upper-body fatness anthropometric measurement for weight management in children and adults^21,22^, due to the associated low cost and feasibility^23,24^. Previous studies observed a strong association between NC, total abdominal fat, SAT and VAT using CT or MRI^17,25,26^. However, the association of a larger NC with a higher IMAT is still to be determined. Still, IMAT determination is limited by the radiation exposure associated with CT and by the relatively high cost of magnetic resonance imaging (MRI) analysis precluding its use at clinical settings. Therefore, simple anthropometric indicators, such as NC, waist circumference (WC), body mass index (BMI) but also fat mass (FM) or fat mas index (FMI) will be more practical to predict cardiovascular risk factors at clinical settings as alternatives to estimate IMAT. Nevertheless, the usefulness of these practical anthropometric measures and indexes of adiposity as surrogates of IMAT at the tissue level is still unclear.

Simple and feasible anthropometric measures such as BMI and WC have been explored as markers of IMAT^27–30^. The usefulness of simple, low cost and feasible anthropometric indicators, such as NC, over other commonly used overall or central fat mass (FM) indexes has not been explored as potential markers of IMAT. Therefore, in the present investigation we aimed to examine the association of NC and other simple measures such as WC, BMI, FM and FMI with thigh IMAT, and VAT measured with computed tomography (CT) in overweight/obese women.

## Methods

### Research design and participants

The baseline data of 142 premenopausal overweight and obese Caucasian women participating in a randomized controlled trial (Clinical Trials, ID: NCT00513084) were used. The inclusion criteria were being >24 years, >24.9 kg/m^2^ BMI, not being pregnant, without any cardio-metabolic disease, not being on any medications or not having had an intervention that affects the weight or body composition. All the assessment was performed in the Faculty of Human Movement, Technical University of Lisbon, from 2002 till 2003. The informed consent was signed by the participants. The study protocol was performed according to the principles of the Helsinki Declaration and approved by the Human Research Ethics Committee of the University of Lisbon.

### Procedures

#### Anthropometry

With the participants in anatomical position, standing or sitting with the head in the Frankfort plane and shoulders relaxed, NC was measured with a plastic tape calibrated weekly over the thyroid cartilage, and perpendicular to the longitudinal axis of the neck, according to International Society for Advancement of Kinanthropometry protocol^31^. Based on test-retest in 10 participants, the coefficient of variation (CV) for the NC was 0.45%. Weight and height were measured using a scale (SECA, Hamburg, Germany) and a stadiometer (Seca, Hamburg, Germany) previously calibrated and WC was measured according to Lohman *et al*.’s^32^ procedures. All measurements were made in duplicate, using the mean for the analysis. BMI was calculated by dividing body weight by the squared height in meters (kg/m^2^).

### Body composition

#### Single cross-sectional CT

Using a CT scan (Somaton Plus; Siemens, Sorheim, Germany), participants were assessed in supine position with arms extended above their head. A single cross-sectional CT at the L4-L5 intervertebral space image was acquired to measure the VAT area. The boundary between VAT and abdominal subcutaneous adipose tissue was defined using the abdominal and oblique muscles in continuity with the deep fascia of the paraspinal muscles and the anterior aspect of the vertebral body^33^. Cross-sectional CT thigh images were also obtained using contiguous 7-mm–thick cross-sectional images of both legs obtained between the inferior ischial tuberosity and the superior border of the patella. The IMAT region was measured between muscle groups and underneath the fascia. The IMAT volume (cubic centimeters) identified in each image was calculated by multiplying the image thickness (7 mm) by the tissue area (square centimeters), and IMAT volume (litters) was then converted to mass units (kilograms) multiplying the volume by the assumed constant of fat density (0.92 kg/L)^34^.

All images were obtained using 120 kVp, 480 mA, and 512 × 512 matrix with a 48-cm field of view. CT data were analysed by specific software (Slice-O-matic, Version 4.2, Tomovision,Montreal, Canada) based on image morphology. A combination of watershed techniques and edge detection filters was employed. Different tissues were identified using boundaries in Hounsfield Units (HU) set to −29 to +150 for muscle, −190 to −30 for IMAT and subcutaneous adipose tissue, and −150 to −50 for VAT^35^.

#### Dual energy X-ray absorptiometry (DXA)

Total fat mass, fat-free mass, lean soft tissue, appendicular lean soft tissue mass, leg fat and trunk fat were measured with a pencil beam mode DXA (QDR-1500 Hologic, Waltham, Mass, USA). The equipment measures the attenuation of X-rays pulsed between 70 and 140 kV synchronously with the line frequency for each pixel of the scanned image. Following the protocol of DXA described by the manufacturer, a step phantom with six fields of acrylic and aluminium of varying thickness and known absorptive properties was scanned to serve as an external standard for the analysis of different tissue components. The same technician positioning the participants performed the scans and executed the analysis according to the operator’s manual using the standard analysis protocol. Based on test–retest using 10 participants, the coefficients of variation (CV) in our laboratory of FM is 2.9%. FMI was calculated as FM in kg divided by squared height in m.

### Physical activity assessment

All participants were asked to use an accelerometer (ActiGraph, GT1M model, Fort Walton Beach, Florida, USA), worn on the right hip near the iliac crest during 7 consecutive days including weekend days^36^. The delivery and reception of the accelerometers, as well the explanation of its use, were personally carried out with the participants (Ward *et al*. 2005). The devices were activated on the first day at 07:00, and data were recorded in 10-s epochs. The device activation and data download were performed using the software Actilife Lifestyle (ver. 3.2). Processing was performed using the software MAHUffe (ver. 1.9.0.3; available at www.mrc-epid.cam.ac.uk) from the original downloaded files (*.dat). For the analyses, a valid day was defined as having 600 or more minutes (10 h) of monitor wear, corresponding to the minimum daily use of the accelerometer^37^. Apart from accelerometer nonwear time (i.e., when it was removed for sleeping or water activities), periods of at least 60 consecutive min of zero activity intensity counts were also considered as nonwear time. The amount of activity assessed by accelerometry was expressed as the number of minutes per week spent in moderate-to-vigorous physical activity (MVPA) using the cutoff values proposed by Troiano^38^ for moderate intensity (2020–5998 counts/min, corresponding to 3–5.9 METs) and vigorous intensity (≥5999 counts/min, corresponding to ≥6 METs).

### Statistical analyses

Descriptive statistics were performed to describe the characteristics of the study participants. Pearson correlation analyses were used to examine the relationship of NC and other anthropometric indicators with thigh IMAT and VAT.

Multivariate lineal regression was performed to examine the unadjusted associations of NC, BMI, WC, FM and FMI with thigh IMAT and VAT indicators (model 1) or to adjust for age (model 2). Statistical analyses were performed using the SPSS software version 21.0 (SPSS, Chicago, IL, USA) and statistical significance was set at P-value < 0.05.

## Results

**Figure 1:**
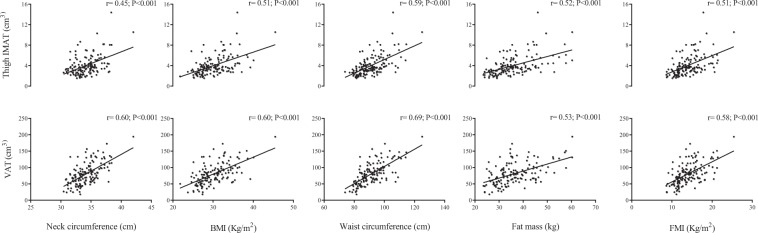
Association of neck circumference (NC), Body mass index (BMI), Waist circumference (WC) and Fat mass index (FMI) with thigh Intermuscular adipose tissue (IMAT) and Visceral adipose tissue (VAT) in premenopausal Women (n = 142). Pearson correlation coefficients and P-value are provided.

Table 1 displays the descriptive characteristics of the participants of the study. The accelerometer wear mean time was 13.8 ± 1.3 h/day and participants spend the 71% of their waking time (16.91 ± 1.5 h/day) in sedentary behavior. Figure 1 illustrates the significant correlations between NC, BMI, waist circumference, FM and FMI with thigh IMAT  and VAT.

**Table 1:** Descriptive characteristics of the study participants.

Variables	N	Mean ± DS
Age (years)	142	38.1 ± 5.8
Weight (kg)	142	78.0 ± 10.8
Height (m)	142	1.6 ± 0.1
NC (cm)	142	34.2 ± 1.9
BMI (kg/m²)	142	30.2 ± 3.8
WC (cm)	142	91.4 ± 8.7
ThighIMAT volume (cm^3^)	142	4.0 ± 2.0
ThighIMAT area (cm^2^)	142	5.8 ± 2.8
VAT (cm^3^)	142	80.8 ± 32.9
VAT (cm^2^)	142	115.4 ± 47.0
Legs fat mass (kg)	142	13.2 ± 3.1
Trunk fat mass (kg)	142	18.0 ± 4.8
Total fat mass (kg)	142	36.2 ± 8.1
Appendicular lean soft tissue mass (kg)	142	16.0 ± 2.3
Total fat free mass (kg)	142	41.2 ± 4.7
Lean soft tissue (kg)	142	38.7 ± 4.4
Waking time (min/day)	109	827.4 ± 75.8
Sedentary time (min/day)	109	1015.2 ± 89.0
Light physical activity (min/day)	109	381.3 ± 84.6
Moderate physical activity (min/day)	109	34.3 ± 19.0
Vigorous physical activity (min/day)	109	1.46 ± 4.1
Moderate-vigorous physical activity (min/day)	109	35.8 ± 20.4

Values of sample size together with mean ± standard deviation, are provided. BMI: Body mass index; IMAT: Intermuscular adipose tissue; NC: Neck circumference; VAT: Visceral adipose tissue; WC: Waist circumference.

In Table 2, the unadjusted (Model 1) or adjusting for age (Model 2) show that NC, BMI, WC, FM and FMI were associated with thigh IMAT and VAT in premenopausal women (all P > 0.01). Values of NC in the unadjusted analyses (Model 1) were positively associated with thigh IMAT, and VAT (Standardized β coefficient: β = 0.45, P-value ≤ 0.001, β = 0.60, P = ≤ 0.001; respectively). When linear regression analyses were additionally adjusted for age, NC remained significantly associated with thigh IMAT (β = 0.41, P = ≤ 0.001). Similarly, NC was a significant predictor of VAT, independently of age (β = 0. 54, P = ≤ 0.001). We repeated the analyses adjusting for height, FM (kg) and MVPA and the results did not change (data not shown).

**Table 2:** Association of neck circumference (NC), Body mass index (BMI), Waist circumference (WC) Fat mass (FM) and Fat mass index (FMI) with thigh Inter-muscular adipose tissue (IMAT)and Visceral adipose tissue (VAT) in premenopausal Women.

N = 142	Thigh IMAT (cm3)		VAT (cm^3^)	
	Model 1	Model 2	Model 1	Model 2
NC (cm)	0.45 **	0.41 **	0.60 **	0.54 **
BMI (kg/m^2^)	0.51 **	0.47 **	0.60 **	0.54 **
WC (cm)	0.59 **	0.56 **	0.69 **	0.64 **
FM (kg)	0.52 **	0.49 **	0.53**	0.49**
FMI	0.51 **	0.47 **	0.58 **	0.52 **

Multivariate lineal regression was performed to examine the association of NC, BMI, WC, FM and FMI with Thigh IMAT and VAT indicators after not adjusting for any confounder (model 1) or adjusting for age (model 2). Standardized β coefficient, and P-value are provided. Statistically significant values are in bold: *P ≤ 0.01, **P ≤ 0.001. BMI: Body mass index, FM: Fat mass, FMI: Fat mass index, IMAT: Intermuscular adipose tissue, NC: Neck circumference, WC: waist circumference, VAT: visceral adipose tissue.

## Discussion

The current investigation shows, for the first time, a positive association of NC with thigh IMAT volume in overweight and obese premenopausal Caucasian women. These findings suggest that NC could be a valid measure of ectopic fat deposition as well as abdominal body fat in overweight and obese premenopausal women.

Our results extend previous findings that underlined (the fact) that NC has as a simple and practical indicator of adiposity^21,39^ and is positively associated with VAT measured with MRI and CT^16,17,25,40^. In addition, we observed that, after adjusting for age, NC, BMI, WC, FM, and FMI remained significant predictors of VAT. Interestingly, NC was similar to other indicators as VAT predictor. As no previous investigations explored the association between NC with thigh IMAT, no comparisons can be provided. However it should be underscored the role of intermuscular depots of fat located in the thigh as a relevant predictor of cardiometabolic risk factors in adults who are normal, overweight or obese^11,41^. Taken together, these findings uncover the potential role of NC as a subrrogate of a patogenic neck adipose tissue due to a greater flow of systemic free fatty acids (FFA), that could partly explain the cardiometabolic risk missed by VAT^17,40^. In fact, previous evidence has showed that elevated FFA concentrations reproduce the metabolic abnormalities of obesity and that the upper body fat increased in women with obesity would be the most important contributor in the systemic FFA release^42^.

In this investigation, we observed that anthropometric indicators and FM markers were correlated with ectopic fat deposition, being this association particularly higher for WC and FM. In additon, we showed that an increase in adjusted BMI, WC, FM and FMI markers explained an increase of 0.5–0.6 units in IMAT, while for NC this assocation had a lower variation (0.45 units of IMAT). These results extend previous observations between anthropometric markers with body fat as the outcome of interest^23,43^. In a sample of overweight and obese adults where body-fat percentage was measured by a bioelectrical impedance analysis (BIA), Joshipura *et al*.^43^ showed a lower coefficient of correlation of NC (r = 0.45, P = <0.001) compared to WC (r = 0.62, P = <0.001) and BMI (r = 0.65, P = <0.001), even after adjusting for age, gender, smoking status, and physical activity. Similarly, Assyov *et al*.^23^ in a sample of adults that included pre and postmenopausal women with obesity found that WC (r = 0.60, P = < 0.001) showed a higher association with adiposity compared to NC (r = 0.43, P < 0.001). Nevertheless, NC presents a better association in the assessment of metabolic health compared to WC, specifically with fasting plasma glucose, fasting insulin, uric acid, HDL cholesterol and serum triglycerides^26,43^. Recently, similar results have been shown by Borel *et al*. in 305 women with severe obesity where NC was a better anthropometric marker to identify a high cardiometabolic risk compared to WC and BMI^18^. The interesting observation is that FMI presented a slightly lower ability to predict VAT and IMAT in the adjusted model compared to BMI. This is in line with Ortega *et al*.’s findings^44^, who showed that BMI was a stronger predictor of CVD mortality than total adiposity markers, particularly, fat-mass percentage and FMI assessed using accurate methods.

It is important to underscore that the reference techniques used in this study to assess IMAT and VAT along with DXA measures for total and regional body composition determination strengthen the findings of this study. Also, the inclusion of accelerometry data to assess the role of habitual physical activity as a potential confounder in these findings was explored. Nevertheless, some limitations should also be addressed. The cross-sectional nature of this study does not establish causality, and a possible reverse causality between anthropometric measures and adipose tissues compartments should not be disregarded. Our findings can only be generalized to Caucasian overweight/obese premenopausal women.

In conclusion, we suggest that a larger NC is associated with a higher volume of VAT but also with a higher amount of ectopic fat deposition in the thigh skeletal muscle, underscoring the relevance of NC as an indicator of adipose tissue content in thigh skeletal muscle. Additionally, when WC, BMI or adiposity are not available or are invalidated (edema, abdominal distension or increase in lean mass) in the clinical practice, NC should be used as an alternative anthropometric measure to predict thigh IMAT and VAT in overweight and obese premenopausal women due to its simplicity, feasibility and low cost.
