# Publisher Correction: Neck circumference is associated with adipose tissue content in thigh skeletal muscle in overweight and obese premenopausal women

**DOI:** 10.1038/s41598-020-69150-4

**Published:** 2020-07-16

**Authors:** Maria Jose Arias Tellez, Analiza M. Silva, Jonatan R. Ruiz, Sandra S. Martins, António L. Palmeira, Teresa L. Branco, Claudia S. Minderico, Paulo M. Rocha, José Themudo-Barata, Pedro J. Teixeira, Luís B. Sardinha

**Affiliations:** 1 Department of Nutrition, Faculty of Medicine, University of Chile, 1027 Independence, Santiago Chile; 2 PROFITH “PROmoting FITness and Health Through Physical Activity” Research Group, Department of Physical and Sports, Education, Faculty of Sports Science, Sport and Health University Research Institute (iMUDS), University of Granada, Ctra de Alfacar s/n, C.P. 18071 Granada, Spain; 3 Exercise and Health Laboratory, CIPER, Faculdade Motricidade Humana, Universidade de Lisboa, Estrada da Costa, 1495-688 Cruz Quebrada, Portugal; 4 Universidade Europeia, Lisbon, Portugal; 5 Faculdade de Medicina, Instituto de Saúde Ambiental (ISAMB), Universidade de Lisboa, Lisbon, Portugal

Correction to: *Scientific Reports* 10.1038/s41598-020-65204-9, published online 20 May 2020.

In the original version of this Article, the author Maria Jose Arias Tellez was incorrectly indexed. This error has now been corrected.

